# Mediating effect of psychological capital on the relationship between mental health literacy and coping styles among newly recruited nurses

**DOI:** 10.1186/s12912-024-01828-w

**Published:** 2024-03-14

**Authors:** Liyuan Xing, Ying Lu, Haixin Zhang, Zhiyi Shi, Shuying Chang, Weihua Liu, Jie Kou, Hongmei Zhang

**Affiliations:** 1 Department of Nursing, Henan Provincial People’s Hospital, No.7, Weiwu Road, 450003 Zhengzhou, China; 2 Department of Nursing, Zhengzhou University People’s Hospital, Zhengzhou, China; 3 Henan Provincial Key Medicine Laboratory of Nursing, Zhengzhou, China; 4 Henan University People’s Hospital, Zhengzhou, China

**Keywords:** Psychological capital, Mental health literacy, Coping style, New nurses

## Abstract

**Background:**

Newly recruited nurses face multiple sources of stress and their coping styles need to be focused on to ensure good mental health. This study aimed to examine the relationship among mental health literacy, psychological capital and coping styles in newly recruited nurses.

**Methods:**

A cross-sectional study was conducted in August and September 2022. A total of 315 newly recruited nurses were recruited in a tertiary hospital in Henan Province, central China, employing the convenience sampling method. The self-reported questionnaires were sent through a QR code, including the Mental Health Literacy Scale for Healthcare Students, Psychological Capital Questionnaire, and Simplified Coping Style Questionnaire. Pearson correlation analysis was used to evaluate the relationships among the variables. Mediation analysis was performed to identify the mediating effect of psychological capital on the relationship between mental health literacy and coping styles.

**Results:**

Positive coping showed a positive relationship with psychological capital and mental health literacy, while negative coping showed a negative relationship with psychological capital and mental health literacy. For positive coping, psychological capital was a partial mediator with an effect of 0.140, accounting for 62.8%. For negative coping, a full mediating effect was shown by psychological capital between mental health literacy and negative coping, with an indirect effect of -0.048.

**Conclusion:**

Psychological capital plays a partial and complete mediating role between mental health literacy and different coping styles among newly recruited nurses. Diversified training and personalized guidance in improving mental health literacy and increasing psychological capital simultaneously can be provided to newly recruited nurses continuously to adjust their coping styles.

## Introduction and background

Occupational stress is a recognized problem in nursing that causes physical, emotional, and mental attrition [1]. According to the literature, nurses experience various stressors such as heavy workloads, conflict at work, role ambiguity, and incidences of aggression [2]. For nurses newly recruited in hospitals, including newly graduated nurses and experienced nurses changed to a new organization, transition shock, assimilation into employment, workplace incivility, new environment and relationships are also experienced [3–7]. According to Lazarus & Folkman’s transactional theory of stress and coping, coping behaviors are activated when people face stress [8]. Two coping styles consisting of positive and negative coping were identified in the literature [9, 10]. Positive coping bolsters and supports an individual’s physical and mental state, while negative coping leads to burnout and psychological distress [11–14]. As the salutogenic model states, health is a dynamic continuum and stress is everywhere, which is both harmful and beneficial [15]. Positive coping with stress can promote people to move toward the healthy pole [16]. Much of the existing research focuses on investigating new nurses’ stressors and coping styles [6, 17–19]. Due to the stressful character of the nursing profession, nursing managers can shift the focus on how to promote positive coping and reduce negative coping, in order to ensure good mental health in newly recruited nurses.

The salutogenic model focuses on understanding and promoting factors that contribute to health and well-being rather than solely on the causes of diseases with a health-oriented perspective [20, 21]. Salutogenesis can be thought of as an ongoing process of learning that promotes health-related behavior by raising health literacy [21]. Health literacy refers to obtaining, understanding, evaluating, and utilizing health-related information and services to make well-informed health decisions [22]. When it comes to mental health, mental health literacy has gained attention and is believed to affect mental health behavior [23]. Mental health literacy is derived from health literacy, and its definition has evolved over these years [24]. As evidence for research on mental health literacy increases, researchers state that the components of mental health literacy include positive mental health, reduction of stigma towards mental illness, the ability to identify common psychological problems and understand the risk factors and causes, and help-seeking efficacy and attitude [23, 25, 26]. Conceptually, mental health literacy can help people understand, apprehend, appraise and apply mental health-related knowledge. A previous study found that mental health literacy is a protective factor in adopting positive coping strategies in adolescents [27]. Therefore, we hypothesize that mental health literacy positively influences positive coping and negatively influences negative coping in newly recruited nurses.

The salutogenic model also points out that coping resources can be identified and mobilized in the process of people facing stress, in which personal psychological resources are one of the most critical factors in general coping resources [28, 29]. Psychological capital was considered a positive psychological resource to maintain a positive mental state [30]. Psychological capital is defined as a positive psychological state shown by individuals in the process of growth and development, composed of four elements: hope (an individual’s belief in working towards a goal and achieving success), resilience (an individual’s confidence in accomplishing something and the spirit of facing difficulties), optimism (a positive prediction of the present and the future) and self-efficacy (an individual’s measurement and evaluation of whether he or she can successfully accomplish something) [30]. As a positive psychological resource, psychological capital has also been demonstrated to influence the coping style, indicating that people with high psychological capital often adopt positive coping styles [31, 32]. Therefore, we infer that mental health literacy can mobilize psychological capital to cope when facing stressors. In other words, we hypothesize that psychological capital mediates the relationship between mental health literacy and coping styles. This study thus used mediation analysis to evaluate the relationships between psychological capital, mental health literacy and coping styles among newly recruited nurses.

## Methods

### Design

We utilized a cross-sectional design and conducted this study in Henan Province, central China, in August and September 2022.

### Participants

Participants were chosen by using convenience sampling in a tertiary hospital of Henan Province in central China and recruited during the period of pre-job training, which provided hospital policy introduction and basic nursing skills training. Considering that some nurses with clinical experience face new environmental and interpersonal challenges when transitioning to a new setting, this study did not exclude them. The inclusion criteria were (1) nurses whom the hospital had newly recruited, and (2) having a smartphone that can complete the questionnaire through the QR code. Nurses who asked for leave during pre-job training and refused to participate in this study were excluded.

### Sample size

We used Monte Carlo power analysis to calculate the sample size [33]. The entered correlations between all variables were obtained from the previous studies: mental health literacy with resilience psychological capital (*r* = 0.586), psychological capital and coping style (*r* = 0.329), mental health literacy and positive coping (*r* = 0.312) [27, 32, 34]. The minimum sample size was 232, with 0.8 power and 95% confidence level. Some participants might answer the questionnaire too quickly or select the same answer for every item. Therefore, 20% more participants, which meant 290 participants, were required to ensure the data quality.

### Data collection

We collected data through an online questionnaire platform. With the help of the hospital manager, we described the purpose of the research, the possible duration, and the precautions for filling out the questionnaire face-to-face in the pre-job training activity for newly recruited nurses. This was an anonymous questionnaire with no personally identifiable information collected. Whether they participated or not would not impact their daily work and welfare. They were then shown the QR code, and nurses willing to participate could scan it to fill in the questionnaire. Informed consent was provided in the first part of the questionnaire and the above content was re-emphasized. Nurses were free to withdraw from the study at any point. The participants only could submit the questionnaire after answering all questions on their smartphones. Four hundred new nurses were recruited by the hospital and participated in the pre-job training. Finally, 315 nurses filled out the questionnaire, with a response rate of 78.75%. All the completion time was more than 5 min. No participant selected the same answer for every item. 315 questionnaires were included in the analysis.

### Measurements

#### Mental health literacy scale for healthcare students (MHLS-HS)

The MHLS-HS, designed by Chao et al., was used to measure the degree of mental health literacy of health professionals and students [35]. The scale includes 26 items across five dimensions: (a) positive mental health maintenance, (b) recognition of mental illness, (c) stigma attitude toward mental illness, (d) efficacy in help-seeking, and (e) attitude towards help-seeking. A 5-point Likert scale was used to score every item, with 1 signifying strongly disagree and 5 strongly agree. The items of the third dimension were reverse scoring. The higher the score, the higher level of mental health literacy. The scale has been tested among healthcare students and has demonstrated good validity and reliability, with 0.81 in Cronbach’s alpha coefficient. In this study, Cronbach’s alpha coefficient of the overall scale was 0.809, while Cronbach’s alpha coefficient for the five dimensions varied between 0.747 and 0.822. The fit indexes of the confirmatory factor analysis were χ2/df = 1.885, CFI = 0.915, TLI = 0.904, RMSEA = 0.067, and SRMR = 0.075, demonstrating good construct validity [36].

#### Psychological capital questionnaire (PCQ-24)

Luthans et al. first developed the PCQ-24 to measure employees’ psychological capital level, with 0.87 in Cronbach’s alpha [30]. The Chinese version of the PCQ-24 was translated and adapted by Li and showed good validity and reliability in nurses [37]. Four positive psychological resources named hope, efficacy, resilience and optimism were assessed by 24 items, with each dimension comprising 6 items. A 6-point Likert scale was used to score the degree of psychological capital, with 1 (strongly disagree) to 6 (strongly agree). Items 13, 20, and 23 were scored in reverse. The higher score indicates a higher degree of psychological capital. In this study, Cronbach’s alpha of this questionnaire was 0.926.

#### Simplified coping style questionnaire (SCSQ)

Xie developed the SCSQ to investigate the most likely adopted attitudes and behaviors when people encounter difficulties or experience setbacks [38]. There were 20 items with two subscales, with 12 items assessing positive coping and 8 items in negative coping. Every item used a 4-point scale ranging from 0 (never used) to 3 (often used), while each subscale was scored separately. Higher scores indicated higher levels in the corresponding coping style when an individual faces difficulties. The Cronbach’s alpha of the scale tested in this study was 0.750, while the two subscales were 0.810 and 0.740, respectively.

### Ethical consideration

The Ethics Committee of Henan Provincial Peoples’ Hospital approved this study (No. 202074). All participants were informed that participation in the study was voluntary and that they could withdraw at any time. Each participant gave their informed consent before they took part in this study. All data were confidential and anonymous and were only accessed by the researchers.

### Data analysis

SPSS IBM 25.0 and SPSS PROCESS macro were used for data analysis. The normality test was performed first and described through mean and standard deviation, stratified by newly recruited nurses whether with nursing practice experience. We used frequency and percentage to present counting data. The independent samples *t*-test was conducted to compare the variables’ scores between newly recruited nurses with no previous work experience and with experience. Pearson correlation analysis was used to evaluate the relationships between these variables in the whole sample: positive coping, negative coping, mental health literacy and psychological capital. We employed mediation analysis to examine whether the mediating variable partially explain the correlation between variables [39]. Model 4 of SPSS PROCESS macro tested the mediating effect, and the bootstrap method with 5000 samples provided confidence intervals (CIs) [40]. We set mental health literacy as the independent variable and psychological capital as the mediating variable, to perform mediation effect analysis with positive coping and negative coping as dependent variables respectively. Based on the results of previous studies, nurses of different ages and lengths of experience coped differently [11, 32, 41]. The ages were significantly different in nurses who whether had previous experience in the present study. Therefore, age and whether had previous work experience were set as covariates in the mediating effects analysis of this study. The result was significant when the 95% confidence interval (CI) did not include a value of 0.

## Results

### Participant characteristics

There were 315 valid questionnaires, comprising 178 with no previous work experience and 137 with experience. Newly recruited nurses with experience were older (mean = 27.21, SD = 3.09) than those with no experience (mean = 22.42, SD = 1.01). The difference was significant (t = 19.408, *p* < 0.001). Among 315 participants, 248 female and 67 male nurses, 16.5% were the only child in their family, and 83.5% were not. 87% were single, and 13.0% were married. Regarding the birth region, 57.8% were born in rural areas and 42.2% in urban areas. The other sociodemographic variables are shown in Table 1.

**Table 1:** Sociodemographic characteristics of participants (*N* = 315)

Variables		NRN with no experience (*N* = 178)	NRN with experience (*N* = 137)	Overall sample (*N* = 315)
		N (%)/M ± SD	N (%)/M ± SD	N (%)/M ± SD
Age		22.42 ± 1.01	27.21 ± 3.09	24.50 ± 3.22
Gender				
	Female	135 (75.8)	113 (82.5)	248 (78.7)
	Male	43 (24.2)	24 (17.5)	67 (21.3)
Marital status				
	Single	176 (98.9)	98 (71.5)	274 (87.0)
	Married	2 (1.1)	39 (28.4)	41 (13.0)
Only child in family				
	Yes	35 (19.7)	17 (12.4)	52 (16.5)
	No	143 (80.3)	120 (87.6)	263 (83.5)
Birth region				
	Rural	106 (59.6)	76 (55.5)	182 (57.8)
	Urban	72 (40.4)	61 (44.5)	133 (42.2)
Health conditions				
	Good	83 (46.6)	68 (49.6)	151 (47.9)
	General	94 (52.8)	67 (48.9)	161 (51.1)
	Bad	1 (0.6)	2 (1.5)	3 (0.9)
Number of Kids				
	None	176 (98.9)	110 (80.3)	286 (90.8)
	One or Two	2 (1.1)	27 (19.7)	29 (9.2)
Satisfaction with own economic conditions				
	Very satisfied	26 (14.6)	22 (16.1)	48(15.2)
	Satisfied	90 (50.6)	67 (48.9)	157(49.7)
	General	59 (33.1)	45 (32.8)	104(33.0)
	Dissatisfied	3 (1.7)	3 (2.2)	6(1.9)
Educational level				
	Bachelor	164 (92.1)	133 (97.1)	297(94.3)
	Diploma	14 (7.9)	4 (2.9)	18(5.7)

NRN = Newly recruited nurses

### Scores of mental health literacy, psychological capital, and coping styles

The independent sample t-test showed that the resilience score in newly recruited nurses with experience (mean = 28.33, SD = 4.11) was higher than those with no experience (mean = 27.24, SD = 4.14). The difference was significant (t = 2.317, *p* = 0.021). However, the magnitude of the difference in the means was minimal (eta squared = 0.017). There were no other significant differences for other dimensions of these variables. The mean scores of positive coping for the whole sample were 27.26 ± 4.79, while those for negative coping were 8.19 ± 3.82. The mean score for mental health literacy for the whole sample was 104.07 ± 9.55, including the mean scores of positive mental health maintenance (42.63 ± 5.24), recognition of mental illness (17.16 ± 2.52), attitude of mental illness stigma (19.85 ± 3.85), help-seeking efficacy (12.13 ± 2.24), and help-seeking attitude (12.29 ± 2.34). The mean score of psychological capital was 113.07 ± 14.96, including the mean scores of efficacy (28.40 ± 4.63), hope (28.37 ± 4.59), resilience (27.71 ± 4.16), and optimism (28.59 ± 3.86). Table 2 presents the scores for coping style, dimensions of mental health literacy, and psychological capital, stratified by newly recruited nurses whether with experience.

**Table 2:** Scores of scales with dimensions

Scales	Range	Overall sample (*N* = 315)		NRN with no experience (*N* = 178)		NRN with experience (*N* = 137)		t	*p*
		M	SD	M	SD	M	SD		
Positive coping	12–36	27.26	4.79	27.24	4.83	27.30	4.75	0.116	0.908
Negative coping	0–20	8.19	3.82	8.28	3.93	8.08	3.69	-0.448	0.654
Mental health literacy (total)	64–128	104.07	9.55	104.29	9.69	103.78	9.39	-0.465	0.642
Maintenance of positive mental health	20–50	42.63	5.24	42.32	5.11	43.04	5.40	1.215	0.225
Recognition of mental illness	5–20	17.16	2.52	17.35	2.46	16.91	2.59	-1.542	0.124
Attitude to mental illness stigma	8–30	19.85	3.85	19.85	4.20	19.85	3.36	-0.004	0.997
Help-seeking efficacy	4–15	12.13	2.24	12.29	2.21	11.93	2.26	-1.439	0.151
Help-seeking attitude	4–15	12.29	2.34	12.47	2.33	12.05	2.34	-1.584	0.114
Psychological capital	71–144	113.07	14.96	112.60	14.83	113.69	15.16	0.641	0.522
Self-efficacy	16–36	28.40	4.63	28.75	4.50	27.94	4.78	-1.534	0.126
Hope	12–36	28.37	4.59	28.33	4.11	28.42	4.78	0.162	0.871
Resilience	15–36	27.71	4.16	27.24	4.14	28.33	4.11	2.317	0.021
Optimism	19–36	28.59	3.86	29.00	3.84	28.28	3.86	1.656	0.099

NRN = Newly recruited nurses

### Correlations between mental health literacy, psychological capital, and coping styles

The whole sample was analyzed to examine the correlations between mental health literacy, psychological capital, and coping styles. The results showed that mental health literacy was positively associated with positive coping (*r* = 0.446, *p* < 0.01), and psychological capital was also positively associated with positive coping (*r* = 0.578, *p* < 0.01). Negative coping was negatively associated with mental health literacy (*r* = − 0.137, *p* < 0.05) and psychological capital (*r* = − 0.221, *p* < 0.01). The results reflected a positive correlation between mental health literacy and psychological capital (*r* = 0.574, *p* < 0 0.01). The correlations among the study variables are presented in Table 3.

**Table 3:** The correlations among positive coping, negative coping, mental health literacy and psychological capital in newly recruited nurses (*n* = 315)

Variables	1	2	3	3.1	3.2	3.3	3.4	3.5	4	4.1	4.2	4.3	4.4
1.Positive coping	1												
2.Negative coping	0.023	1											
3.Mental health literacy (total)	0.446^**^	-0.137^*^	1										
3.1Maintenance of positive mental health	0.506^**^	-0.06	0.764^**^	1									
3.2Recognition of mental illness	0.151^**^	0.001	0.484^**^	0.282^**^	1								
3.3Attitude to mental illness stigma	-0.017	-0.175^**^	0.426^**^	0.032	-0.112^*^	1							
3.4Help-seeking efficacy	0.284^**^	-0.112^*^	0.570^**^	0.255^**^	0.191^**^	0.08	1						
3.5Help-seeking attitude	0.278^**^	-0.032	0.601^**^	0.275^**^	0.268^**^	0.065	0.459^**^	1					
4.Psychological capital	0.578^**^	-0.221^**^	0.574^**^	0.548^**^	0.172^**^	0.135^*^	0.399^**^	0.325^**^	1				
4.1Self-efficacy	0.515^**^	-0.205^**^	0.528^**^	0.463^**^	0.151^**^	0.129^*^	0.416^**^	0.341^**^	0.874^**^	1			
4.2Hope	0.539^**^	-0.154^**^	0.513^**^	0.504^**^	0.171^**^	0.074	0.371^**^	0.304^**^	0.920^**^	0.780^**^	1		
4.3Resilience	0.511^**^	-0.181^**^	0.454^**^	0.446^**^	0.145^*^	0.124^*^	0.286^**^	0.222^**^	0.885^**^	0.691^**^	0.761^**^	1	
4.4Optimism	0.433^**^	-0.234^**^	0.493^**^	0.491^**^	0.125^*^	0.147^**^	0.298^**^	0.251^**^	0.781^**^	0.515^**^	0.624^**^	0.619^**^	1

^*^p < 0.05, ^**^p < 0.01

### Mediating effects

**Fig. 1:**
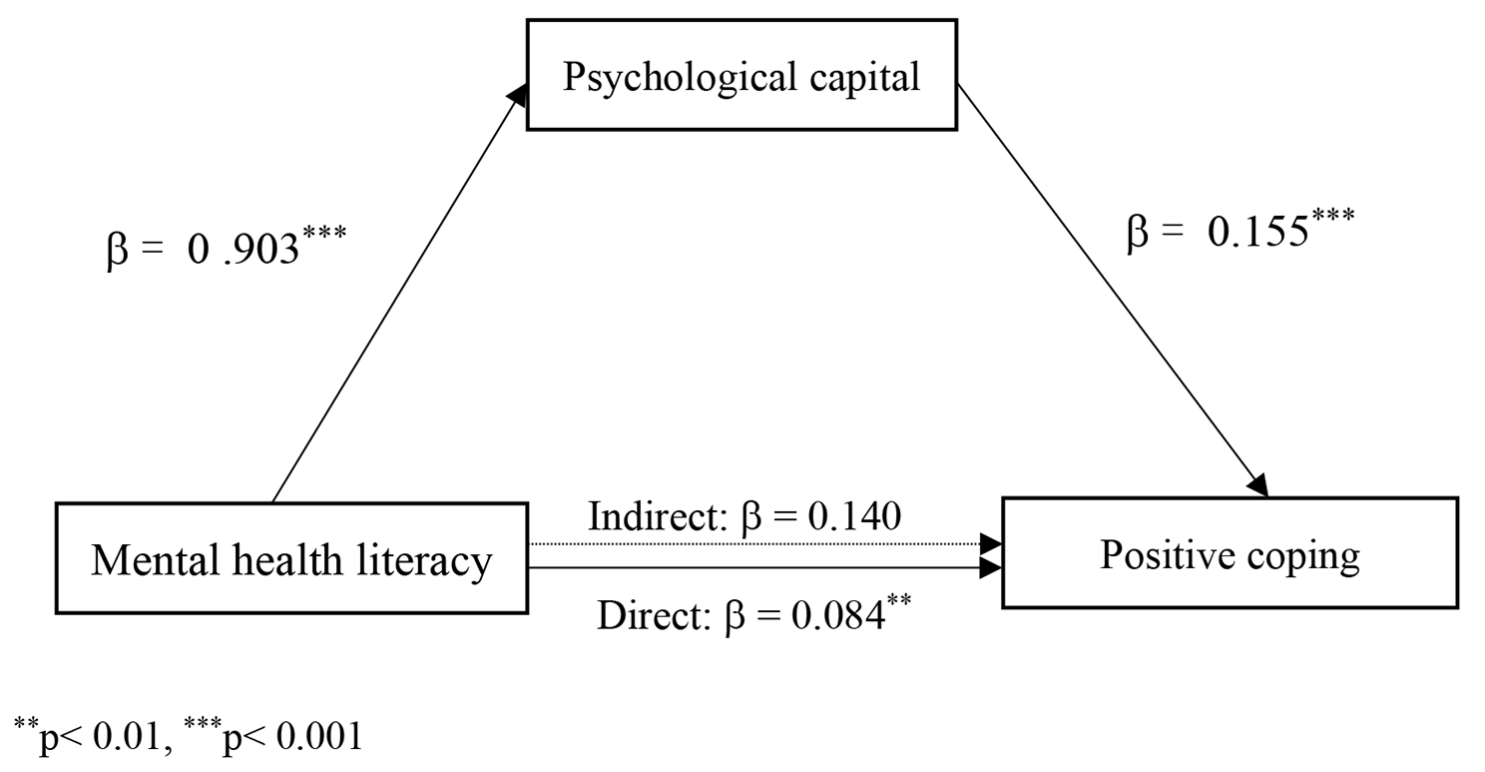
Mediating effects of psychological capital on the relationship between mental health literacy and positive coping

**Fig. 2:**
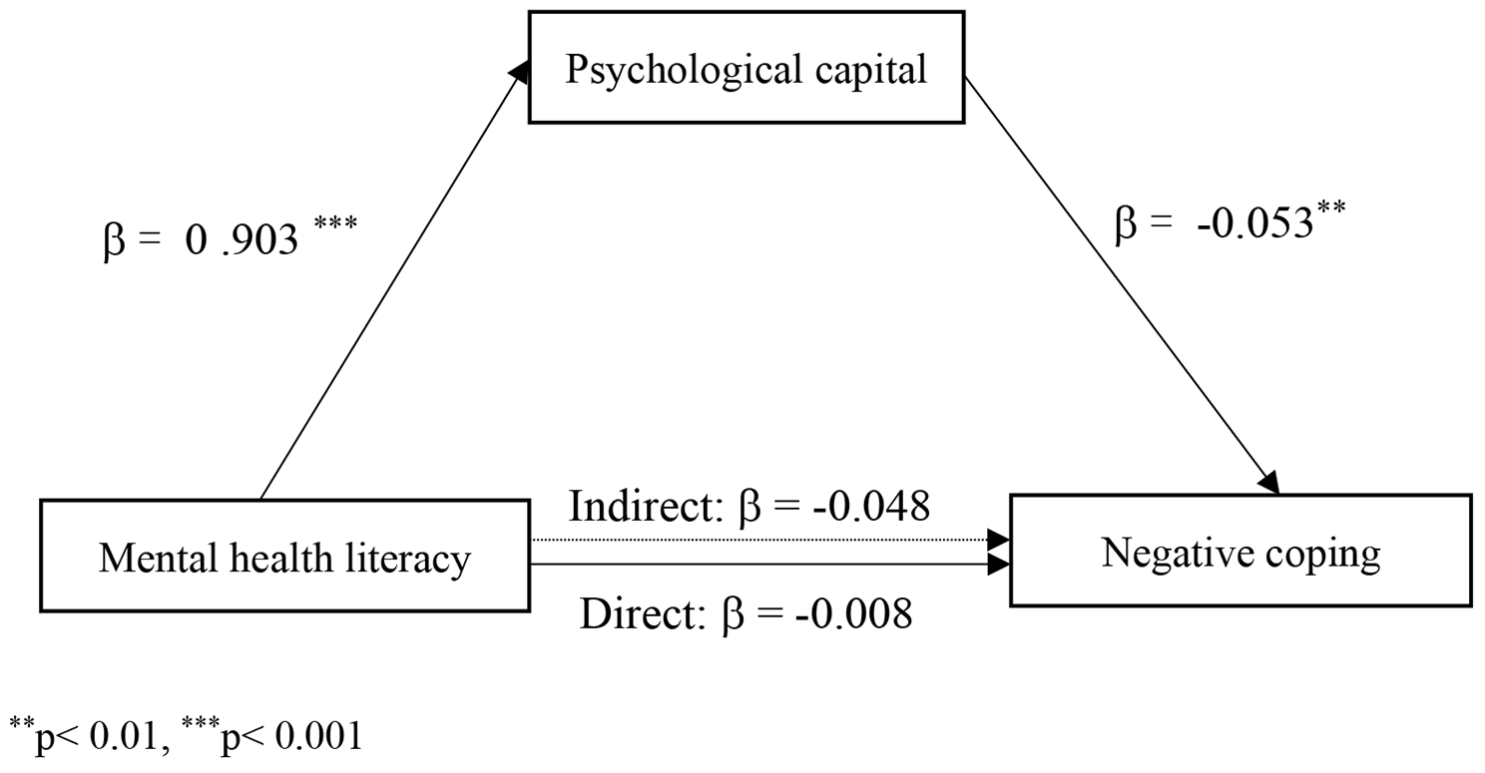
Mediating effects of psychological capital on the relationship between mental health literacy and negative coping

When examining mediation effects, the whole sample was analyzed. The results showed that psychological capital partially mediates the relationship between mental health literacy and positive coping. The mediating effect of psychological capital on the relationship between mental health literacy and positive coping was 0.140, accounting for 62.8% of the total effect, and the bootstrap BC 95% CI was 0.101–0.183 (see Fig. 1). When analyzing the mediating effect of psychological capital in the relationship between mental health literacy and negative coping, a fully mediating role was found, with an indirect effect of − 0.048 and a bootstrap BC 95% CI of − 0.078– − 0.017 (see Fig. 2). The mediation analysis results are presented in Table 4.

**Table 4:** Mediating effects of psychological capital on the relationships between mental health and coping style

Indirect effect	Effect of X on M	Effect of M on Y	Direct effect	Indirect effect	Total effect	Effect size	Bootstrapping (BC 95%CI)	
							Lower	Upper
Mental health literacy (X) → psychological capital (M) → positive coping (Y)	0.903 ^***^	0.155^***^	0.084^**^	0.140	0.223^***^	0.021	0.101	0.183
Mental health literacy (X)→ psychological capital (M)→ negative coping (Y)	0.903^***^	-0.053^**^	-0.008	-0.048	-0.056^*^	0.015	-0.078	-0.017

^*^p < 0.05, ^**^p < 0.01, ^***^p < 0.001

## Discussion

The coping style of newly recruited nurses needs attention, as it determines the impact of stress on an individual’s mental health and the nursing workforce. Therefore, this study investigated mental health literacy, psychological capital, and coping styles among newly recruited nurses, including nurses with work experience but transitioning to a new setting, based on the salutogenic model. When comparing these variables’ scores in nurses whether with experience, only the resilience score showed a statistically significant difference (*p* = 0.021). However, the eta squared of 0.017 suggested that it might not have practical significance [42]. Then we performed the subsequent analyses on the whole sample.

We found a positive correlation between mental health literacy and positive coping and a negative correlation between mental health literacy and negative coping. This is similar to the results in a sample of teachers [43]. A high level of mental health literacy represents more knowledge and beliefs about identifying risk factors, managing pressure, and seeking appropriate help [25]. Therefore, nursing managers should emphasize the importance of mental health literacy in newly recruited nurses. It should be noticed that the dimension of positive mental health maintenance was most related to positive coping (r = 0.506, p < 0.01), followed by help-seeking efficacy and attitude (r = 0.284, 0.278, p < 0.01, respectively). This finding was consistent with those of Carvalho and Vale-Dias [27]. Positive mental health maintenance is the central component of mental health literacy, which helps people to deal with stressful situations well and master the surroundings with efficacy [26]. We also found that negative coping was negatively associated with attitudes to mental illness stigma and help-seeking efficacy (*r* = − 0.175, *p* < 0.01; *r* = − 0.112, *p* < 0.05). Although the correlation coefficient was low, this result provided a significant reference value for developing corresponding interventions to reduce the negative coping of newly recruited nurses indirectly. The hospital can add mental illness-related knowledge education to the pre-job training to improve the correct cognition of mental health disorders and reduce the related stigma. Diversified psychological support resources can also be provided and publicized from the hospital level to improve their help-seeking efficacy.

In addition, this study shows that psychological capital has a mediating effect on the relationship between mental health literacy and coping styles. Mental health literacy represents people’s knowledge and attitudes toward psychological problems or high-pressure environments, and coping is how people behave in these situations [23, 44–46]. The data illustrate that mental health literacy does not simply impact positive coping through the mediation of psychological capital (β = 0.084; *p* < 0.01) but also directly affects positive coping (β = 0.140). When testing the mediating effect of psychological capital in the relationship between mental health literacy and negative coping, we found that psychological capital was a full mediator in such a relationship (β = − 0.048). The results suggest that mental health literacy positively influenced psychological capital (β = 0.903; *p* < 0.01), which again negatively affected negative coping (β = − 0.053; *p* < 0.01). As a positive resource, nurses with higher psychological capital could adjust their cognition to positively cope with the stressor and alleviate negative coping with an optimistic and confident attitude [47, 48]. Similarly, a previous study also reported the full mediating role of psychological capital between stress and coping styles in nurses working in the intensive care unit [49]. The finding of this study indicates that nurse managers and policymakers can support newly recruited nurses through these two routes [50, 51]. First, a variety of education modalities to increase mental health literacy can be offered continuously after recruiting nurses in the hospital, especially the promotion of positive mental health maintenance and help-seeking efficacy and attitude, and the reduction of stigma attitude towards mental health disorders, which can be considered essential insights to improve coping styles. In addition, targeted training or personalized guidance can be provided at the organizational level according to the attributes of psychological capital to increase positive psychological resources among newly recruited nurses.

This study has several limitations. Firstly, it is worth noting that psychological capital fully mediates the relationship between mental health literacy and negative coping style, but the effect size is relatively small. This does not rule out the existence of other mediators [7, 52, 53]. Further research with multi-center large sample data will be needed for testing the possible mediators in the future. Secondly, the cross-sectional design could not infer causal relationships. Thirdly, the generalization of our results should be performed with caution because of the bias of the one-center study and the convenience sampling method. Finally, all data were self-reported using questionnaires to collect data. Therefore, future studies can be performed at multiple centers to increase the model’s generality. Longitudinal studies should also be conducted to study changes in nurses’ coping styles.

## Conclusion

This study evaluated the relationships and interactions among mental health literacy, psychological capital, and coping styles based on the salutogenic model. Psychological capital was a mediating variable, playing a partial and complete mediating role between mental health literacy and different coping styles among newly recruited nurses. Our results suggest developing interventions from two routes of mental health literacy and psychological capital, to continuously improve positive coping style among newly recruited nurses. Nurse managers and policymakers should provide mental health-related education and diverse training to enhance mental health literacy, especially promoting positive mental health maintenance and help-seeking efficacy and attitude, and the reduction of stigma attitude towards mental health disorders. Meanwhile, psychological capital should be developed among newly recruited nurses. Considering that the mediating effect of psychological capital on mental health literacy and negative coping is relatively small, further research could explore other potential mediators.

## Data Availability

The raw data supporting the conclusions of this article will be made available by asking the corresponding author.
